# Targeting mTOR and eIF4E: a feasible scenario in ovarian cancer therapy

**DOI:** 10.20517/cdr.2021.20

**Published:** 2021-05-11

**Authors:** Alice Romagnoli, Cristina Maracci, Mattia D’Agostino, Anna La Teana, Daniele Di Marino

**Affiliations:** ^1^Department of Life and Environmental Sciences, Polytechnic University of Marche, Ancona 60131, Italy.; ^2^New York-Marche Structural Biology Center (NY-MaSBiC), Polytechnic University of Marche, Ancona 60131, Italy.

**Keywords:** Ovarian cancer, targeted therapy, mTOR pathway, eIF4E, inhibitors

## Abstract

Ovarian carcinoma is one of the most common causes for cancer death in women; lack of early diagnosis and acquired resistance to platinum-based chemotherapy account for its poor prognosis and high mortality rate. As with other cancer types, ovarian cancer is characterized by dysregulated signaling pathways and protein synthesis, which together contribute to rapid cellular growth and invasiveness. The mechanistic/mammalian target of rapamycin (mTOR) pathway represents the core of different signaling pathways regulating a number of essential steps in the cell, among which protein synthesis and the eukaryotic initiation factor 4E (eIF4E), the mRNA cap binding protein, is one of its downstream effectors. eIF4E is a limiting factor in translation initiation and its overexpression is a hallmark in many cancers. Because its action is regulated by a number of factors that compete for the same binding site, eIF4E is an ideal target for developing novel antineoplastic drugs. Several inhibitors targeting the mTOR signaling pathway have been designed thus far, however most of these molecules show poor stability and high toxicity *in vivo*. This minireview explores the possibility of targeting mTOR and eIF4E proteins, thus impacting on translation initiation in ovarian cancer, describing the most promising experimental strategies and specific inhibitors that have been shown to have an effect on other kinds of cancers.

## INTRODUCTION

Ovarian cancer ranks 5th among the most common causes of cancer death in women worldwide^[1]^. The American Cancer Society estimates that in 2021 about 21,410 new cases of ovarian cancer will be diagnosed and about 13,770 women will die from ovarian cancer in the United States^[2]^. With its overall 5-year survival rate of 30% for the advanced stage disease^[3,4]^, ovarian cancer has the worst prognosis and the highest mortality rate among gynecologic cancers^[5,6]^. This is due to lack of early diagnosis tools and to the fact that the earliest symptoms are easy to overlook, as they can be confused with other common illnesses. Symptoms usually become more severe by the time the tumor has spread to the surface of the peritoneal cavity, making it much harder to remove by surgical intervention. Indeed, the standard approach in ovarian cancer therapy is surgery, followed by platinum-based chemotherapy, in which cisplatin or carboplatin is combined with taxanes^[7-9]^.

Despite the initial efficacy of treatment, more than 65% of patients relapse and develop acquired resistance to platinum-based chemotherapy^[10,11]^. Platinum-based drugs, such as cisplatin, interact with DNA and form intra- or inter-strand DNA cross-links, thereby activating cell death but also DNA repair pathways^[12-14]^. Despite the large number of studies^[15,16]^ and the evidence of the multiple mechanisms of sensitivity and resistance to platinum agents^[17]^, there is an urgent need to target additional mechanisms underlying ovarian cancer platinum-resistance.

Drug resistance is the outcome of the deregulation of several molecular mechanisms, such as drug inactivation, apoptotic stimulation, expression of pro-survival or anti-survival proteins, and alteration of the expression of growth factor receptors^[18-21]^. For the treatment of cisplatin-resistant ovarian cancer, a few options are available with the need to improve treatment efficacy and reduce toxicity^[10,22,23]^.

To overcome these limitations, new analogs of conventional drugs and new therapeutic options are being developed, among which several are currently being tested in clinical trials and others have recently been approved^[24,25]^; however, overcoming drug resistance constantly requires new molecular targets that may provide, in addition to conventional treatment, a more selective therapeutic approach to fight ovarian cancer. To overcome drug resistance and formulate customized individual therapies to improve early diagnosis, research needs to focus on the molecular background of ovarian cancer^[11]^. Translation and signaling pathways represent ideal molecular targets for the development of novel therapeutic strategies. Protein synthesis regulates every aspect of cell phenotype, growth, and metabolism and is tightly controlled by several signaling pathways in response to different external stimuli^[26]^. In human cancer, the dysregulation of these processes has an impact in overall protein synthesis, leading to cancer development and growth^[27]^.

This minireview focuses on the recent research reporting the use of inhibitors of the mechanistic/mammalian target of rapamycin (mTOR) and the eukaryotic translation initiation factor 4E (eIF4E) as adjuvants in cancer treatment; despite the limited number of studies performed on ovarian cancer cell models, the encouraging results obtained *in vitro* and in pre-clinical studies with some inhibitors might establish the use in ovarian cancer therapy.

## THE mTOR PATHWAY: A POTENTIAL THERAPEUTIC TARGET

The AKT/mTOR pathway is crucial for the regulation of transcription, translation, cell growth, motility, survival, proliferation, autophagy, and angiogenesis^[28,29]^. Not surprisingly, this pathway is largely deregulated in cancer, where several genes coding for proteins of this axis are frequently mutated, leading to pathway hyperactivation in disease. The majority of the cancer-associated mutations occur in the mechanistic/mammalian target of rapamycin (*mTOR*) gene^[30]^. Because it is found to also be upregulated in ovarian carcinoma^[31,32]^, and its deregulation has been associated with platinum-drug resistance^[33,34]^, mTOR represents an attractive biological target for ovarian cancer therapy.

**Figure 1:**
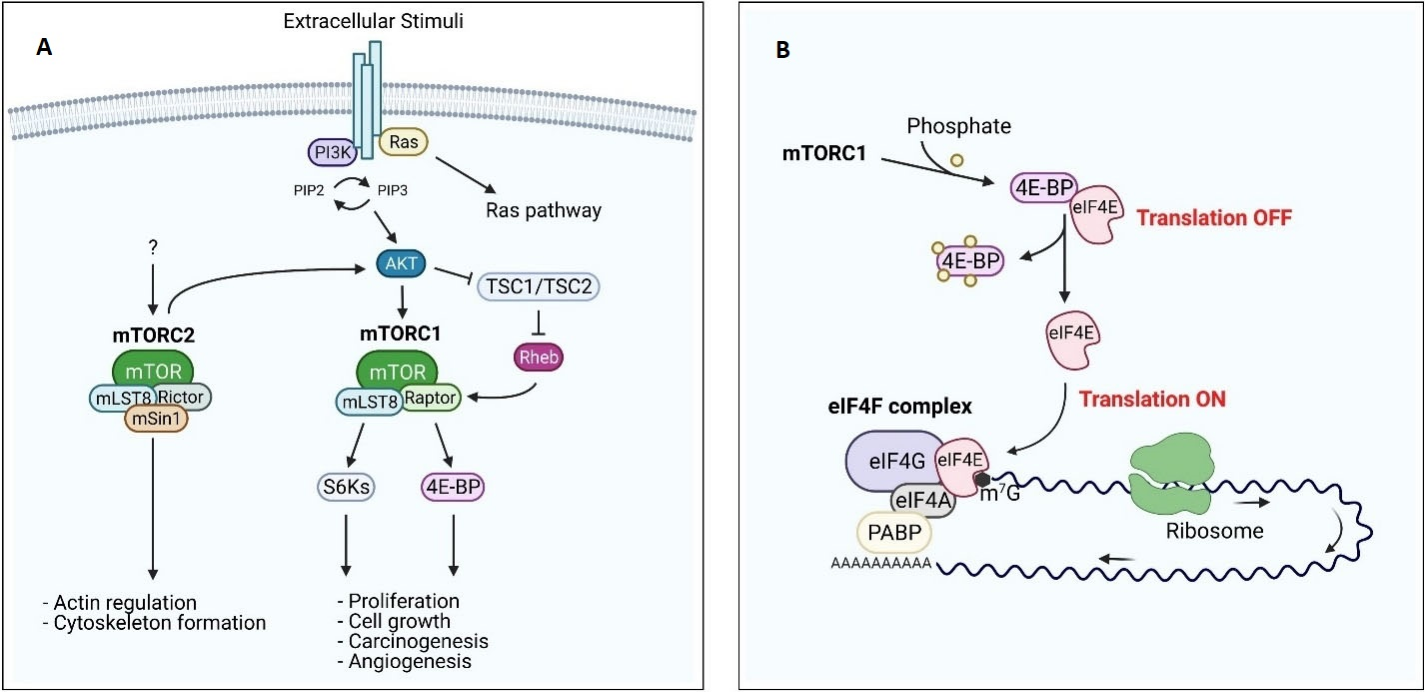
PI3K/mTOR pathway and regulation of eIF4F complex. (A) PI3K and its downstream effectors AKT and mTOR are activated by many extracellular stimuli. The serine/threonine protein kinase mTOR forms, by association with several binding partners (mLST8, Raptor/Rictor, and mSIN1), 2 distinct complexes mTORC1 and mTORC2. mTORC2 activation leads to actin regulation and cytoskeleton organization, while mTORC1 allows the cap-dependent translation through the 4E-BPs phosphorylation, resulting in the dissociation of these proteins from eIF4E. PI3K pathway dysregulation results in an altered proliferation, carcinogenesis, and angiogenesis. (B) The cap-binding protein eIF4E is released by 4E-BPs as a result of its phosphorylation by mTORC1, allowing eIF4F complex formation and thus permitting translation of eIF4E-sensitive mRNAs.

mTOR is a 289-kDa serine/threonine kinase of the phosphatidylinositol 3-kinase related kinase (PIKK) family. In mammals, mTOR is the core component of two functionally different multi-proteins complexes: mTOR Complex 1 (mTORC1) and mTOR Complex 2 (mTORC2). mTORC1, or rapamycin-sensitive complex, includes the proteins mTOR, mLST8 (or GβL), and Raptor (regulatory-associated protein of mTOR) and plays a key role in the regulation of mRNA translation and cell growth^[31,35]^. mTORC2 (rapamycin-insensitive complex), consisting of mTOR, mLST8, Rictor (rapamycin-independent companion of mTOR), and mSin1 (or mitogen-activated protein-kinase-associated protein 1), is mainly involved in actin cytoskeleton dynamics [Figure 1A]^[31,35]^. mTOR is a downstream mediator in the phosphatidylinositol-3-kinase (PI3K)/AKT pathway; in fact, as a consequence of extracellular stimuli, PIP2 is phosphorylated to produce PIP3 by PI3K. This leads to the activation of the protein kinase B (AKT) via phosphorylation by phosphatidylinositol-dependent kinase 1 and 2 (PDK1 and PDK2)^[36]^. Activated AKT phosphorylates mTOR on Ser2448, or indirectly by phosphorylation of tuberin protein or tuberous sclerosis complex 2 (TSC2). TSC2 is complexed to TSC1, and, when active, its function is to convert Rheb (Ras homolog enriched in brain)-GTP to Rheb-GDP, inactivating mTORC1. Once phosphorylated, TSC2 loses its affinity for TSC1, leading to activation of mTORC1^[29,36]^. Activated mTORC1 phosphorylates the translation regulating factor ribosomal S6 kinase-1 (S6K-1) and the eukaryotic translation initiation factor 4E (eIF4E) binding family of proteins (4E-BPs) [Figure 1A]. The phosphorylation of S6K-1 leads to the translation of ribosomal proteins, elongation factors, and other proteins involved in the cell cycle, while phosphorylation of 4E-BPs causes the release of eIF4E [Figure 1B]^[37,38]^. eIF4E is a crucial translation initiation factor, also involved in the onset of a number of cancer types (see discussion below)^[39,40]^.

Contrary to mTORC1, the mechanism by which mTORC2 is activated remains elusive. PI3K stimulates mTORC2 by responding to growth factors. Activated mTORC2 is found to be associated to ribosomes^[41]^, and it directly activates AKT, through phosphorylation of Ser473. This, in turn, activates mTOR, protein kinase C-α, and serum- and glucocorticoid-induced protein kinase 1, acting on actin regulation, as well as on metabolism and cell survival^[29,36]^.

It is clear that the mTOR signaling pathway has an important role in malignancy, as it is frequently hyperactivated in a wide range of tumors, including ovarian cancer^[11,42]^. As a consequence, it represents an attractive candidate for drug design. To date, several inhibitors developed against different proteins of AKT/mTOR pathway are already available at different stages in clinical research, as shown in Table 1^[39,43]^. The best characterized mTOR inhibitor is rapamycin (or sirolimus), a macrolide produced by the bacterium *Streptomyces hygroscopicus*. Rapamycin binds selectively to mTORC1 interacting with a specific binding domain present in mTORC1, but not in mTORC2. Rapamycin inhibits the serine/threonine kinase activity of mTORC1 with an allosteric mechanism^[43]^. Despite the activity shown by rapamycin in many tumor types^[29,44]^, its clinical use is unsuccessful because of its poor water solubility^[29]^. To overcome this limitation, several analogs of rapamycin, called rapalogs, have been formulated, some of which show encouraging pharmacological features on several cancers. Among rapalogs, there are CCI-779 (temsirolimus), RAD001 (everolimus), and AP23573 (deforolimus)^[43,45-49]^. Some rapalogs are FDA-approved, while others are still in clinical trials^[40,43]^. However, the therapeutic efficacy of rapalogs is hindered by the appearance of negative feedback loops that trigger the activation of AKT in the mTOR pathway^[29,39]^. Second-generation inhibitors are thus being developed, and many of them are already under clinical trial. These compounds have shown promising preliminary results, as dual-specificity inhibitors (targeting both mTOR and PI3K) and inhibitors targeting directly mTOR, inhibiting both mTORC1 and mTORC2^[37,39,43] ^[Table 1]. Among the downstream effectors of mTOR, the p70S6 kinase is also implicated in fundamental cellular processes, such as cell growth and proliferation^[38]^. p70S6k, together with 4E-BPs and eIF4E, is frequently activated in a wide range of cancer types and could be a driver or malignancy, also for ovarian cancer^[50-52]^. Thus, targeting p70S6K using specific inhibitors already developed represents another alternative strategy against rapamycin-resistant tumors^[53]^.

**Table 1:** Main inhibitors of mTOR and eIF4E

**Target**	**Category**	**Compound**	**Clinical trials in ovarian cancer**	**Ref.**
mTORC1	Antibiotic	Rapamycin		[54]
	Rapalogs	Temsirolimus	Completed	[45,46]
		Everolimus	Phase I/II	[47,48]
		Deferolimus		[49]
mTORC1 and mTORC2	Second generations inhibitors	Torin1		[55]
		INK128		[56]
		AZD8055		[57]
		AZD2014	Phase I/II	[58]
PI3K and mTOR	Dual inhibitors	PI-103		[59]
		NVP-BEZ235	Completed	[60]
		SF1126		[61]
		GNE-477		[62]
		XL765		[63]
*eIF4E* expression	Antisense oligonucleotides	ISIS 183750		[64]
		LY2275796		[65]
	miRNA	miR-768-3p		[66]
eIF4E phosphorylation	MEK inhibitor	U0126		[67]
	Small molecule	CGP57380		[68]
	Natural product	cercosporamide		[69]
eIF4E-partners interactions	Nucleoside analog	Ribavirin		[70-78]
	Small molecules	4Ei-1		[79,80]
		4EGI-1		[81,82]
		4E1RCat		[83]
	Oligopeptides	GnRH-4EBP fusion peptide		[84]
		4E-BP mimetics peptides		[85-87]

## TARGETING mTOR PROTEIN IN OVARIAN CANCER

On the basis of the promising activity of these compounds on other cancer types, mTOR inhibitors have been tested on ovarian cancer^[35,36,88]^. Rapamycin treatment of several ovarian cancer cell lines resulted in a decrease in the phosphorylation levels of mTOR and 4E-BPs, together with an increase of p-AKT^[42]^. These effects lead to an accumulation of eIF4E-4E-BPs complexes, which blocks protein translation and inhibits ovarian cancer cells proliferation^[42]^. Despite the above-mentioned promising results, rapamycin’s clinical use is currently restricted due to adverse bioavailability^[36]^. The rapalogs Temsirolimus and Everolimus have been tested in clinical trials, showing anti-proliferative and antiangiogenic actions on ovarian cancer, especially when administrated in combination with carboplatin and paclitaxel^[35,36]^. However, as found in other cancers, the critical limitation of these inhibitors is the occurrence of resistance during the treatment, with a molecular mechanism that remains elusive. Loss of the negative feedback loops associated to PI3K/AKT/mTOR might trigger drug resistance mechanisms, one of which concerns the activation of mTORC2 that in turn activates AKT in response to mTORC1 inhibition^[35]^. In addition, differences in AKT activity observed in different ovarian cancer cell lines contribute to the cell-specific sensitivity to pharmacological treatment by rapamycin and, more in general, by mTOR inhibitors^[16]^. In fact, as mentioned above, AKT signaling is the central hub of cellular proliferation and growth as well as activates alternative pathways^[29]^. Besides molecules able to target both mTORC1 and mTORC2 complexes, AZD2014 and AZD8055 have been tested in different ovarian cancer models, showing antitumor activities in combination with others drugs^[57,58]^.

Testing the effects of natural products in ovarian cancer therapy is of clinical interest. Resveratrol (3,4’,5-trihydroxy-trans-stilbene) is a polyphenol found in several plants and wines and thus naturally present in the human diet^[89]^. It has been shown to have anti-neoplastic effects on several types of carcinomas^[90]^, likely via inhibition of hypoxia-inducible factor 1α (HIF-1α) and vascular endothelial growth factor^[91]^, both of which are expressed at higher levels in many human cancers, including ovarian cancer^[11,92]^. The mechanisms of inhibition by resveratrol are multiple; among them, its interference in the AKT/mTOR pathway, leading to a phosphorylation of S6K1 and 4E-BPs. Resveratrol also increases apoptosis and autophagy and decreases proliferation and invasiveness of ovarian cancer cells^[93,94]^.

Further strategies in the development of anti-cancer compounds targeting ovarian malignancies include the suppression of proteins that affect the mTOR cascade, using siRNA^[95]^, or the inactivation of AKT, PI3K, or simultaneous block of mTOR and PI3K, designing dual mTOR/PI3K inhibitors. Simultaneously targeting 2 kinases belonging to the same signaling pathway should lead to a more efficient inhibition of the PI3K/AKT/mTOR cascade and lower the probability of developing drug resistance. In addition, these compounds are developed to prevent AKT hyperactivation due to rapamycin or rapalog treatment^[88]^. The most clinically relevant dual drugs are PI-103, SF1126, GNE-477, XL765, and NVP-BEZ235, the latter being the only one that has been tested in a clinical trial^[35,36,60,88]^. *In vitro* and *in vivo* studies in ovarian cancer models have shown that these inhibitors decrease tumor cell growth and proliferation, also in combination with paclitaxel^[96-98]^. Interestingly, all these findings show that platinum resistance of ovarian cancer cells can be reversed by inhibiting mTOR (mTORC1/mTORC2), as specific mRNAs encoding survival, cell cycle, and other functions are inhibited^[57,99]^.

However, despite the promising results obtained targeting the mTOR pathway, there is a constant need to hunt for new molecular targets and therapeutic approaches. Therefore, the downstream mediator of mTOR pathway, the cap-dependent translation factor eIF4E, represents a valid candidate, being also a confluence point of multiple pathways.

## eIF4E INHIBITION: THE FUTURE OF OVARIAN CANCER THERAPY?

Translation of proteins promoting cancer progression and invasiveness, such as kinases, transcription factors, and vascular growth factors, is mediated by the mRNA 5’-cap-binding complex, a heterotrimeric protein complex consisting of eIF4E, eIF4G, and eIF4A, which recruits the translation apparatus to the mRNA [Figure 1B]^[37]^. eIF4G acts as a scaffold protein, binding both the mRNA helicase eIF4A and eIF4E, while the function of eIF4E is to bind the 5’ 7-methylguansine (m^7^G) mRNA cap, thereby selecting the mRNAs to be translated. Under normal conditions, eIF4E is expressed at low levels and is therefore the limiting factor in the formation of the cap-binding complex. Overexpression of eIF4E is, however, a signature of several aggressive cancer types, including triple-negative breast cancer and ovarian cancer^[100-106]^. eIF4E undergoes phosphorylation at Ser209 by the mitogen-activated protein kinase-interacting kinases 1 and 2 (MNK1/2), which target the eIF4F complex^[107,108]^. Phosphorylation of eIF4E seems to have no functional role under physiological conditions^[43,109]^ but is an independent prognostic factor in several types of cancer^[110]^. The activity of eIF4E is modulated by a heterogeneous group of 4E-BPs, phosphoproteins that compete with eIF4G for the same binding site on eIF4E. When phosphorylated by the mTORC1 pathway, the affinity of 4E-BPs for eIF4E decreases, facilitating translational initiation [Figure 1B]^[37]^. Because of its tight control, a 2.5-fold increased expression of eIF4E is enough to induce transformation, metastatic progression, and suppression of apoptosis^[102,111]^. Conversely, more and more studies have shown that decreasing eIF4E levels in the cell reduces cell growth and invasiveness in several models of cancers^[112-116]^. It is therefore of utmost importance to develop drugs that hamper the activity of eIF4E by either reducing its expression and phosphorylation levels or competing with its biological partners, namely the m^7^G cap, eIF4G, and the 4E-BPs [Table 1].

Because the activity of eIF4E is strictly dependent on its interaction with the mRNA cap, m^7^G analogs represent attractive drug candidates. By mimicking the mRNA cap structure, m^7^G analogs compete for eIF4E binding, thereby decreasing the rate of translation initiation. Ribavirin, an anti-viral drug commonly used to treat hepatitis C, is a guanosine ribonucleoside analogue, initially characterized as cap-mimetic^[76]^, but this is controversial^[77,78]^. It was tested against acute myeloid leukemia^[71,72]^, ribavirin showed promising results in several cancer models, including breast and ovarian cancer^[74,75,117]^. Notably, even though eIF4E levels are not homogeneous among ovarian cancer cell lines and tissues, ribavirin treatment reduced the growth and survival of ovarian cancer cell lines and increased the efficacy of cisplatin treatment both *in vitro* and *in vivo*^[74]^, an observation that raises the possibility of combining it with other drugs for ovarian cancer therapy. Following the encouraging results obtained with ribavirin, several molecules extracted from m^7^G analog libraries have been selected for further studies. Among them, 7-benzyl guanosine monophosphate (Bn7GMP)^[118]^ and 4Ei-1^[119]^ are currently being studied *in vitro* and *in vivo* and might represent ideal adjuvants in ovarian cancer therapy^[79,80,118,119]^.

The balance between active and inactive eIF4E is controlled by the relative abundance of 4E-BPs and eIF4G in the cell. Thus, to reduce the levels of eIF4E, drug design should focus on finding potential ligands able to outcompete cellular eIF4G and/or mimic the 4E-BPs. 4EGI-1, a small molecule discovered through high-throughput screening of compound libraries^[81]^, is an allosteric eIF4E inhibitor which binds eIF4E in a different region with respect to eIF4G^[82]^, inhibiting eIF4G-eIF4E complex formation and facilitating the binding of 4E-BP1^[120]^. Furthermore, several short peptides have been designed, based on the sequence of eIF4E binding partners, to specifically destabilize the eIF4E-eIF4G complex in the cell, representing a valuable approach to target eIF4E-mediated translation in cancer models^[86,87,121,122]^. The increasing evidence that eIF4E targeting slows down cancer progression is having a prompt response in ovarian cancer research. Recently, a novel strategy to specifically target eIF4E activity in ovarian cancer models has been reported, where 4E-BP1 peptides were fused to an agonist of the gonadotropin-releasing hormone (GnRH)^[84]^, thereby mediating the uptake and showing a marked decrease in tumor cells growth.

Taken together, these studies suggest that eIF4E represents a promising candidate for the research on ovarian tumors, whereas downregulating eIF4E expression might be a useful and feasible approach to improve the therapeutic responsiveness of ovarian cancer. Future research effort must be employed in designing and testing potential drugs against eIF4E, to evaluate the possibility of a cancer-specific drug.

## CONCLUSION

Patients diagnosed with ovarian cancer have poor prognosis, owing to the advanced status of malignancy at the time of diagnosis and the development of resistance to the standard therapy treatment. Thereby, new therapeutic strategies are urgently needed. The mTOR signaling pathway is frequently overactivated in ovarian cancer and converge to the increased levels of cap-dependent translation, being eIF4E the focal point of this pathway. Thus, specific inhibitors targeting mTOR and eIF4E represent promising and valid adjuvants for clinical management of ovarian cancer.

## DECLARATIONS

### Copyright

© The Author(s) 2021.
